# Cognitive interviewing to assess and adapt three measures of mental health symptoms among people living with HIV in Rakai, Uganda: the Thinking a Lot Questionnaire, the Patient Health Questionnaire 9 (PHQ-9), and the Hopkins Symptoms Checklist (HSCL)

**DOI:** 10.21203/rs.3.rs-4697900/v1

**Published:** 2024-07-10

**Authors:** Nora S West, Lydia P Namuganga, Dauda Isabirye, Rosette Nakubulwa, William Ddaaki, Neema Nakyanjo, Fred Nalugoda, Sarah M Murray, Caitlin E Kennedy

**Affiliations:** University of California San Francisco; Johns Hopkins University; Rakai Health Sciences Program; Rakai Health Sciences Program; Rakai Health Sciences Program; Rakai Health Sciences Program; Rakai Health Sciences Program; Johns Hopkins Bloomberg School of Public Health; Johns Hopkins Bloomberg School of Public Health

**Keywords:** Cross-cultural validation, Psychological distress, Cognitive interviewing, HIV/AIDS, Mental health screening tools

## Abstract

Mental health is conceptualized differently across cultures, making cross-cultural validation of screening tools critical. In Uganda, we used cognitive interviewing to assess and adapt three scales for measuring psychological distress: the Thinking a Lot Questionnaire, the Patient Health Questionnaire 9 (PHQ-9), and the Hopkins Symptoms Checklist (HSCL). We recruited 12 people living with HIV from the Rakai Community Cohort Study (RCCS) and interviewed seven potential users of the scales (four RCCS survey interviewers and three local health workers). Data were analyzed systematically using a team-based matrix approach. The HSCL was generally well understood, with minor clarifications needed. The Thinking a Lot Questionnaire was also well understood, though differences between “how much” and “how often” required specificity. Both included local idioms of distress from prior adaptations. The PHQ-9 performed less well, with many questions interpreted variably or showing unclear local applicability, especially among people living with HIV. For example, questions about trouble concentrating were misunderstood, focusing on examples like newspapers rather than the broader issue of concentration. Future research should explore the validity and utility of commonly used instruments as mental health research expands in Africa.

## Introduction

Psychological distress among people living with HIV is common, often goes unrecognized, and has deleterious impacts on HIV treatment outcomes and transmission. Poor mental health and HIV stigma are prevalent and linked to risk behaviors, delayed care seeking, decreased medication adherence, and poor retention in care (Brandt 2009; Chambers et al. 2015; Chibanda et al. 2014; Croome et al. 2017; Mayston et al. 2012; Musisi et al. 2014; Parcesepe et al. 2018; Rueda et al. 2016; Shubber et al. 2016; Wagner et al. 2014). Importantly, mental disorders occur more frequently among people living with HIV than the general population (Brandt 2009; Chibanda et al. 2014; Narayan et al. 2014; Parcesepe et al. 2018).

In Uganda, depression symptom estimates among people living with HIV range from 8–47%, and anxiety disorder symptoms range from 1–28%, with variation partially due to differences in study design and instrument used (Ashaba et al. 2018; Kaharuza et al. 2006; Kinyanda et al. 2011; 2012; Nakimuli-Mpungu et al. 2012; Psaros et al. 2015). Studies from Uganda have demonstrated that depression is linked to both inconsistent condom use and skipped doses of antiretroviral therapy (ART), underscoring the critical need for enhanced mental health support to improve outcomes in HIV treatment (Kinyanda et al. 2018; Musisi et al. 2014). However, these studies are of limited utility to inform practice because they have primarily employed cross-sectional designs that preclude causal interpretations and have predominantly used instruments to assess mental health that have not been validated among people living with HIV in Uganda, meaning estimates of burden and associations with outcomes could be erroneous. In addition, much of the available data on symptomology of poor mental health come from urban and peri-urban populations, while data from rural populations are lacking.

To yield reliable and valid data, mental health measurement instruments must be relevant to local experiences of mental illness and wellness and use appropriate local terms for symptoms (Bass et al. 2007; Kirmayer 1989). Across cultures, mental health is conceptualized, manifested, and expressed in diverse ways, calling into question the validity of measures of mental health that have been developed without first exploring what mental health means and how psychological distress presents in a population to be studied (Chibanda et al. 2015; Summerfield 2008). In Uganda, several exploratory qualitative studies have found symptomology that differs from that measured in mental disorder-specific tools developed in high-income countries (Betancourt et al. 2014; Bolton et al. 2004; Wilk and Bolton 2002). In 2020, we conducted a qualitative study in Rakai, Uganda, to understand how psychological distress is manifested and described for people living with HIV in the region (West NS Unpublished). Health workers identified depression and anxiety as common issues experienced by people living with HIV, but these were rarely mentioned by people living with HIV themselves. Instead, people living with HIV described two local idioms of distress, *okweraliikirira* (worry/apprehension) and *okwenyamira* (deep thoughts/thinking too much), as common problems experienced by people living with HIV in the area.

Cognitive interviewing is a technique used to understand individuals’ cognitive processes when interpreting and responding to survey questions (Willis 2005). We selected three scales for measuring psychological distress based on our formative work: the Thinking a Lot Questionnaire, the PHQ-9, and the Hopkins Symptoms Checklist (HSCL). We performed cognitive interviewing to pre-test the instruments and explore comprehension, clarity, acceptability, recall, response processes, and appropriateness of items and response categories in the Rakai setting.

## Methods

### Study setting

This research was conducted in the Rakai and Kyotera regions of south-central Uganda under the auspices of the Rakai Health Sciences Program (RHSP). RHSP conducts the Rakai Community Cohort Study (RCCS) – an ongoing open, population-based cohort of approximately 18,000 participants per survey round (Chang et al. 2016). To date, mental health instruments have not been included in RCCS surveys. HIV prevalence in Uganda is 5.1% nationally (UNAIDS 2017), and higher in the predominantly rural areas covered by the RCCS, where median prevalence ranges from 14%–42% (Chang et al. 2016; Grabowski et al. 2014).

### Participant recruitment

We recruited two participant groups for cognitive interviews: people living with HIV targeted by the questionnaires and those who may use these instruments in data collection or clinical settings, such as RCCS survey data collectors and healthcare providers. We selected potential respondents from previous RCCS participants who consented to be re-contacted and were 18–49 years old, Luganda-speaking, diagnosed with HIV, aware of their HIV status, and able to give verbal consent. We excluded those previously interviewed in the study’s formative phase. RCCS data collectors and health workers were recruited on RHSP staff recommendations, were 18 or older, Luganda-speaking, currently employed by RHSP, and able to give verbal consent.

### Mental Health Instruments

From our formative work (West NS Unpublished), we identified “thinking too much,” depression, anxiety, and stress (worry/apprehension) as the most potentially relevant manifestations of psychological distress to measure among people living with HIV in this setting. We selected three existing instruments for assessment and potential adaptation. For “thinking too much,” we selected the Thinking a Lot questionnaire (^(Hinton DE et al. 2015)^. At the time of the study, the three-item Thinking a Lot questionnaire was the only instrument for assessing” thinking too much” idioms. The Thinking a Lot questionnaire asks whether participants experience “thinking a lot” in the past four weeks and then assesses how much (*0: not at all, 1: a little, 2: some, 3: much, 4: extremely*) and how often (*0: never, 1: one to two times a month, 2: three to four times a month, 3: two to three times a week, 4: almost every day or daily*) using five-point frequency scales. For depressive symptoms, we selected the nine-item depression module of the PHQ (PHQ-9),(Kroenke et al. 2001) given its widespread use in Uganda and inclusion as the recommended screener for people living with HIV in the Ugandan National HIV Guidelines (Government of Uganda 2018). The PHQ-9 consists of nine items for depressive symptom criteria using a four-point Likert scale to assess how often respondents have been bothered by problems in the last two weeks (*0: not at all, 1: several days, 2: more than half the days, 3: nearly every day*), and includes a question to screen for suicidality. To further explore depression and anxiety with an instrument already adapted to the Ugandan context, we selected the Hopkins Symptom Checklist (HSCL) depression and anxiety symptom inventory (HSCL-25) (Derogatis et al. 1974b). Originally a 25-item measure of anxiety and depression, we used a Luganda translation that was previously adapted and validated among HIV-affected adults and resulted in the inclusion of five additional questions measuring locally identified idioms of distress (Bolton 2001; Bolton et al. 2003). The HSCL-25 scale uses a four-point frequency scale to assess the presence of depression and anxiety symptoms in the past (*1: not at all,2: a little,3: quite a bit, 4: extremely*).

For the PHQ-9, we obtained Luganda versions from two sources: an RHSP study on neurocognition (Sacktor et al. 2019) and a validation and diagnostic accuracy study (Nakku et al. 2016). These two separate Luganda translations of the PHQ-9 differed significantly. The study team reviewed both Luganda versions side by side and discussed translations to arrive at a final version (West NS Unpublished). For the Thinking a Lot questionnaire, we followed WHO guidelines for instrument translation and adaptation (World Health Organization). The questions were translated into Luganda by a bilingual RHSP research staff member, followed by a review meeting with RHSP research staff to assess the translation’s accuracy, discuss the suitability of word choices and conceptual clarity, and resolve any linguistic discrepancies.

### Data collection and analysis

Due to COVID-19 restrictions, all recruitment and interviewing (March-December 2022) were conducted by telephone. Trained RHSP qualitative data collectors contacted potential participants using a script, informed them about the study details, and obtained oral consent. Interviews were scheduled at times convenient for participants, ensuring privacy and availability of a charged phone. The interviews, conducted with a semi-structured qualitative guide, went question by question through either the Thinking a Lot questionnaire and PHQ-9 or the HSCL and explored comprehension, clarity, acceptability, recall, response processes, and appropriateness of the items and response categories for the administered instruments. All participants were also asked about the overall length of the instruments. Only prior RCCS participants were asked to answer the instrument questions, as we sought data collectors’ and health workers’ opinions as professionals engaged in administering the instruments. Instrument administrators were asked for their thoughts about the overall utility of the instruments as part of survey data collection and clinical care. Interviews lasted approximately 60 minutes each.

Cognitive interviewing is an iterative process often involving multiple rounds (Willis and Artino 2013). Initially, 16 participants were interviewed using the first versions of the instruments; nine received the PHQ-9 and “Thinking too much” questionnaire, and seven received the HSCL. The interviews led to refinements in the PHQ-9, which was then retested with three additional RCCS participants. Throughout the process, data collectors summarized preliminary findings and field notes, which were regularly discussed in team meetings to determine when data saturation was reached (Kerr et al. 2010). For the two-part analysis, an analytic matrix (Appendix 1) was developed for each instrument. Organized by measurement instrument and item (e.g., question) for each screener, the matrix summarized participant interview responses and narrative for providing responses and instrument interpreting items, including categories for how the participant determined their answer response, recall of the information asked in the item, ease and comprehension of the item, participant explanation of the item in their own words, and interpretation of specific terms. In the first part of the analysis, L.N., fluent in Luganda and English, completed the analytic matrix using both transcripts in English and audio recordings of the interviews conducted in Luganda. N.S.W. reviewed and finalized the analytic matrix using transcripts in English. The first part of the analytic process was used to refine the instrument between rounds of interviewing and compared across iterative rounds to look for improvement in subsequent instrument applications. In the second part of the analysis, the analytic matrix was used to compare findings across participant types (instrument administrators and people living with HIV) and reviewed findings from additional interviews utilizing the revised instruments and items. We also conducted two different team retreats with Ugandan and US-based team members to review findings question by question in each survey instrument and discuss decisions around item adaptation going forward.

### Ethical considerations

As part of the cognitive interviewing process, RCCS participants were referred for further assessment and support if they met thresholds for mild depression on the PHQ-9 (score ≥ 5), stress on the HSCL (score ≥ 14), or significant distress from “thinking too much” (score ≥ 5). Participants showing endorsing suicidality on the PHQ-9 or HSCL were contacted by specialized mental health staff for emergency support and referral.

This study was reviewed and approved by the Uganda Virus Research Institute (UVRI) Research Ethics Committee, the Johns Hopkins Bloomberg School of Public Health (BSPH) Institutional Review Board, and the Uganda National Council on Science and Technology (UNCST).

## Results

Among the 12 participants living with HIV recruited as potential future respondents to the scale, the majority were female (n = 7) and had primary education (n = 6) or secondary education (n = 4), while two reported no formal education. The seven instrument administrators were majority male (n = 4), all with some post-secondary education. Cognitive interview findings and instrument revisions are detailed by instrument, with final versions in Appendix 2.

### Instrument 1: The Hopkins Symptom Checklist (HSCL)

Overall, the adapted HSCL was well understood by participants, and most said it was easy to determine an answer to the questions:

The question was easy for me based on how you asked it and depending on how I am feeling. If I was feeling low energy in my body, I would tell you that my body is weak. However, now that is not the case.

Answer options (“Not at all”, “Rarely”, “Sometimes, “Often”) were comprehensible to most participants, though some noted complexity of the Luganda language when discussing the frequency of symptoms:

*I think these options make sense. However, the answer option ‘sometimes’ is similar to the answer option ‘often’. ‘Sometimes’: it is not something that is [occurring] regularly, or it is not a regular verb. But when you say, ‘I often go to mosque every day’, it means that you went to the mosque four times. Although I feel like there was some contradiction between “sometimes” and “often”, it is still fine. From what you mentioned, “sometimes” is “*oluusi n’oluusi*”, and then ‘often’ it occurs at regular intervals. For instance, ‘we often go to church every Sunday.’*

When asked about ease of use and time needed to administer the HSCL in either clinical care or during the RCCS, instrument administrators felt that, overall, the number of questions in the instrument was appropriate. However, some suggested that the HSCL might require a skilled interviewer who understood the content of the questions if participants needed further explanation:

It depends on the person [participant] that you are going to interview and the person [interviewer] going to ask them, because questions can look to be so many if the person going to ask them does not understand them, but they are not so many.

After analysis, the study team made no adaptations to the HSCL.

### Instrument 2: The “Thinking Too Much” Questionnaire

Overall, participants comprehended and easily determined their responses to whether they had been bothered by thinking too much or deep thoughts in the timeframe (the past four weeks) specified.

What I understood is that in that time frame mentioned of 28 days, equivalent to one month, how much have I been bothered or how many times have I been bothered by deep thoughts

The main challenge participants raised was perceived similarities in the content of the second and third questions, which ask about how much someone was bothered by “thinking too much” and how often someone was bothered by “thinking too much”, respectively:

Those last two questions you asked about are the ones that can confuse a little. They confused me as well not until you repeated for me the question. Those two questions seem to be asking for the same thing. But when you ask the question again you feel there is a little difference. One is specific and the other is general. So, those are the questions that seem to be similar, but their answer options are not similar. The answer options are different.

The questionnaire was modified to clarify the wording to convey the intention of frequency for the third question, from *“Mu weeks four eziyise, otawanyizidwa emirundi emeka embeera ey’okuloowoza ennyo oba okubera mu birowoozo ebingi?”* to *“Mu weeks ennya (mu weeks ennya nteegeeza okuva leero nga okutuuka nga omwezi oguwedde), Ekyokutawaanyizibwa embeera eyokulowooza ennyo oba okubeera mu birowoozo ebingi, kizze kiddiringana emirundi emeka?”*

### Instrument 3: The Patient Health Questionnaire-9 (PHQ-9)

Five questions (Q2, Q3, Q4, Q5, Q9) were generally well understood by potential respondents and potential instrument users and did not require any modifications. However, the remaining four questions (Q1, Q6, Q7, Q8) presented challenges with interpretation or cultural relevance.

**Question 1: “** *Over the last 2 weeks, how often have you been bothered by any of the following problems: Little interest or pleasure in doing things”*

Potential instrument administrators generally well understood the question; however, participants living with HIV struggled with the intent of the question. Participants living with HIV varied in their reports of what they understood the question to intend to ask about, from how physical illness affects work to experiencing mood changes, and to general trouble doing daily activities as described by a participant:

In my own words, the question is asking me whether I have encountered challenges while doing some work, and I have responded to you, ‘not at all.’

Further, some participants struggled with the answer options, noting that the distinction between “not at all” and “several” days (options 1 and 2) was significant and difficult to delineate in Luganda.

This item was kept the same as the wording was clear to the data collectors, and the variability in participant interpretations did not point to a specific re-wording pathway. However, among the three participants living with HIV who completed interviews using the revised PHQ-9, Q1 persisted as challenging to answer consistently, with participants explaining the question as “barriers or difficulties” faced, worry about “not completing work or tasks,” or feeling “disturbed” by something else in their lives.

**Question 6**: *“Over the last 2 weeks, how often have you been bothered by any of the following problems: Feeling bad about yourself — or that you are a failure or have let yourself or your family down?”*

About half of participants, both instrument administrators and people living with HIV, struggled to comprehend the intent of the question, with a variety of explanations for how they arrived at their answer choice and how they understood the question and its meaning. Descriptions of the meaning of the question ranged from participants assessing whether they had achieved their personal financial goals, feeling like a failure because of circumstances not related to mental health, feeling frustrated, or not having worked enough or made enough money to support their families in the time frame asked, as described by a participant:

I chose “not at all” because like I told you, I have not failed at anything. It has been easy [to answer] because I fulfil all my responsibilities and care for my family members under my care and it is the reason why I swiftly responded to you.

Many participants acknowledged feeling like one “let their family down” would, in turn make one feel bad about oneself, and often focused on the component of the question that talks about letting family down in the context of personal and financial responsibility, as described by a participant:

I would tell [explain to] the person that the question is asking about how he feels about self and his usefulness towards other people whether family members, co-workers, or other people he lives with.

Overall, the item was lengthy when translated into Luganda. The translation was revised to reduce length while retaining the intent and English language structure of the question. Additionally, the translation of “feeling bad about yourself” was adjusted to clarify that the intent was to focus on emotion rather than the physical. Adjustments to Q6 were adequate based on participant responses in the second round of interviews with the revised instrument.

**Question 7**: *“Over the last 2 weeks, how often have you been bothered by any of the following problems: Trouble concentrating on things, such as reading the newspaper or watching television?”*

Many participants focused heavily on the examples of reading the newspaper or watching television. These examples shaped answer choices and interpretation of the question. Some participants noted not reading the newspaper or having access to television factored into their responses. Most participants living with HIV focused on the newspaper and television aspects of the question. They did not factor in the element of “concentration” into their response, as described by a participant:

I understood that question as you asked me regarding reading newspapers or watching television and I told you, I watch television for a short while like the news hour and therefore I have no problem at all.

Most data collectors were attentive to the “concentration” component and clear on the intent of the question overall.

“Radio” was added to the examples of “newspaper or watching television” to make the item more culturally appropriate as many people in this part of Uganda may not have access to a television or read the newspaper. At the same time, radio is a common source of information and entertainment. Given that many participants, particularly people living with HIV, omitted the concept of “concentration” from their cognitive process when responding to the question, the translation of concentration was revised from “*Okufuna obuzibu mu kussaayo ebirowoozo mu by’okola okugeza nga okusoma empapula z’amawulire oba okulaba TV”* to *“okutaataaganyizibwa mu birowoozo ng’olina by’okola okugeza nga okuwuliriza radio, okulaba TV oba okusoma empapula z’amawulire*” to simplify, highlight, and clarify the concept of concentration. Despite these edits, the intent continued to be challenging to understand in the second round of interviews, with participants still focusing on the examples (television, newspaper, radio) and not the concept of concentration.

**Question 8**: *“Over the last 2 weeks, how often have you been bothered by any of the following problems: Moving or speaking so slowly that other people could have noticed? Or so fidgety or restless that you have been moving a lot more than usual?”*

Most participants struggled to explain the full question in their own words. Typically, participants selected one part of the question (e.g., moving or speaking slowly or being fidgety or restless) when formulating their response and explanation, regardless of whether they endorsed experiencing the item as having bothered them. Some participants also linked their response to a physical ailment (e.g., an illness or being unwell), as described by a participant living with HIV:

I have never experienced any problem that would not allow me to walk normally in the past 14 days. For example, I have not suffered any sickness that would not allow me to move normally in those 14 days

Participants who did not focus on both parts of the question in their explanation generally only focused on the first element of “moving or speaking slowly,” as described by an instrument administrator:

This question is trying to focus on whether I have been speaking slowly, or I have not responded to the expectation of someone. For instance, the person may expect my quick response, but unfortunately, I do respond slowly.

The question was ultimately unchanged because when probed, participants understood the meaning of both components of the question despite focusing on the components most relevant to them in their responses.

### PHQ-9 Answer Options

“not at all, several days, more than half the days, nearly every day” and Reflections

Overall, participants felt that the answer options were distinct and understandable. Study staff noted that the translation of “several days” (*ennaku ntonotono*) back translates to “few days” in English. Still, the alternative translation would make it indistinguishable from “more than half the days”.

Despite noted challenges to specific questions, some participants, people living with HIV, mentioned that they felt responses to the questions would help a health worker understand if they or someone else needed additional support or attention, as described by a participant living with HIV:

All the questions are helpful. Actually, if I was experiencing some problems, I think the health workers, after knowing about it, would come and offer help.

Instrument administrators felt that the number of questions was acceptable. Some questions could require additional explanation from whomever was administering or time for the participant to think about their answer. They also noted that participants may select or focus on only certain parts of some questions, as many are long when translated to Luganda.

## Discussion

**Figure 1:**
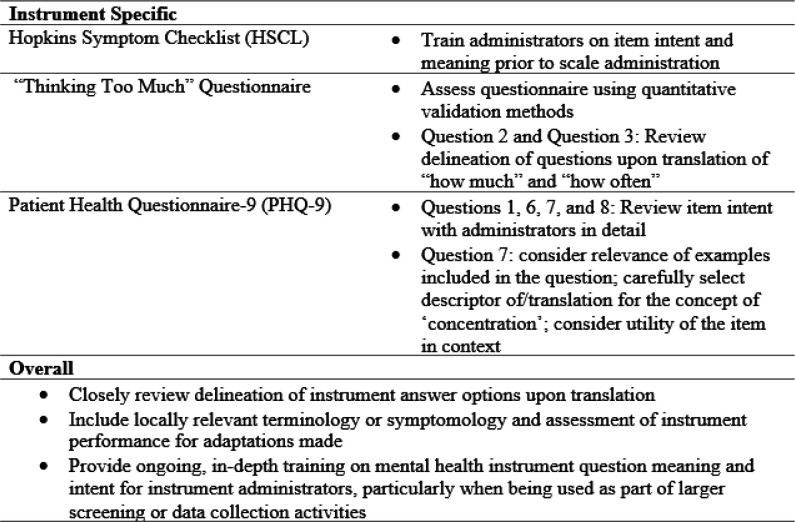
Recommendations to improve cultural relevance and measurement accuracy

This qualitative study explored comprehension of questions and answer options in the Luganda language for three mental health screening instruments among potential future respondents living with HIV and future instrument administrators in research and clinical practice. The HSCL, previously cross-culturally adapted in Uganda and the Luganda language to include local idioms and terms for distress (Bolton 2001) was well understood. The adapted Thinking a Lot questionnaire was generally well understood, with minor adjustments required for the Luganda language. The PHQ-9, a widely used screener for depression, contained four questions (Q1, Q6, Q7, Q8) that were challenging for participants to comprehend and/or answer, two (Q1 and Q7) of which remained difficult for participants to cognitively access when iteratively updated and administered in a second round of interviews. Key recommendations by the instrument are summarized in Fig. 1.

Findings from this study point to the potential impact of language and translation on the utility, meaningfulness, statistical validity, and findings of mental health instruments. Statically validated mental health instruments are important for standardized measurement in the context of diagnostic frameworks, such as the Diagnostic and Statistical Manual of Mental Disorders (DSM). However, the findings of this study demonstrate the importance of considering the utility and meaningfulness of such measures in the local setting. The version of the HSCL used for this study, which was previously adapted for the Ugandan setting and the Luganda language using qualitative research (Wilk and Bolton 2002), was statistically validated among people living with or affected by HIV, with good performance (Ashaba et al. 2018; Bolton 2001). The PHQ-9 has been validated in Luganda, with performance ranging from satisfactory to modest (Miller et al. 2021; Nakku et al. 2016). Screeners typically used in research and practice with people living with HIV, including the PHQ-9 and HSCL, are designed to capture DSM-defined symptoms of mental health (Derogatis et al. 1974a; Kroenke et al. 2001). We adapted the Thinking a Lot questionnaire for this study based on our formative work identifying local idioms of distress, reflecting the descriptions and experiences of people living with HIV and it has yet to be statistically validated in Luganda. Our study offers insights that highlight the complexity of relying on statistical validation as a sole metric and explores the meaning of items and, ultimately, resulting data based on item content and responses. During the planning process we found two different translations of the PHQ-9 questionnaire in Luganda being used in research settings (Nakku et al. 2016; Sacktor et al. 2019), suggesting that the process of translating the items in the instrument is subject to the interpretation of the translator and the complexity of the Luganda language for which single words or concepts in the English language, such as the word and domain of “concentration”, do not have a direct translation in Luganda. Similarly, we found that additional clarification was sometimes required in the translation from English to Luganda to explain that the question was asking about the mental or emotional vs. the physical, such as the concept of “feeling bad about yourself” (Q6). This could be particularly relevant for people living with a chronic illness like HIV, who may think about such questions in the context of illness. While it is likely not feasible to interrogate mental health instruments in the context of every existing health condition and illness, awareness of how underlying illnesses and diagnoses such as HIV, diabetes, tuberculosis, and others may influence responses should be taken into consideration by practitioners and researchers (Akhaury and Chaware 2022; Ninnoni et al. 2023). Further, a study looking at how post-partum women living with HIV in Malawi understand the PHQ-9 and Edinburgh Postnatal Depression Scale (EPDS) found that a number of concepts in the items were not clearly comprehended in the Chichewa language, despite previous statistical validation and a process of translation and back translation (Harrington et al. 2021; Psaki and Hindin 2016). In our study we did not encounter similar challenges with interpretation using the HSCL, likely because we used an already adapted version. While limited, extant literature exploring the topic of language and mental health demonstrates that there are concepts that are not well captured when translating from one language to another (Harrington et al. 2021). The implications of inaccurate mental health measurement in research and practice are considerable, as accurate measurement is tied to individual and community well-being, allocation of resources, global public health guideline recommendations, and prioritization of need.

Beyond the challenges of translation, our findings point to the influence of local context and culture on the interpretation of items and mental health instruments. Participants in our study found the adapted HSCL and “Thinking too much” questionnaire relatively easy to comprehend and answer. “Thinking too much” is a common idiom of distress found across different cultures and contexts (Kaiser et al. 2015) and emerged as common manifestation of psychological distress in our formative work (West et al. 2022; West NS Unpublished). However, “thinking too much” as an idiom of distress varies in its use and meaning across contexts, further highlighting the need for its exploration in the Ugandan context and Luganda language (Kaiser et al. 2015). The Luganda version of the HSCL also includes this idiom of distress, which was added during a local adaptation along with other terms identified by Luganda speakers. For the PHQ-9, which currently contains no local Luganda idioms of distress, there were domains of depression that were clear and well understood by participants including hopelessness, sleep, feeling tired, appetite, and suicidality. The items that persisted as challenging even after revisions focused on decreased concentration (Q7) and loss of interest/pleasure (Q1). For Q7, participants focused on the examples given (reading the newspaper or watching TV) and almost entirely discounted the element of “concentration” in the question. Removing examples from this question or further rephrasing to emphasize the focus of the question is about the ability to concentrate may help participants grasp the intent of the question. For Q1, participants in our study often focused on whether they could do work/perform necessary activities due to some disturbance or issue in their lives. This may point to a cultural and economic emphasis in the context of our study that centers around the ability to work and provide for family, with less focus on leisure activities. Further rewording and revisions to clarify the intent of the question in a way relevant to daily life in this setting are likely warranted to generate answers that map onto the intent of the question.

Instrument administrators in our study raised the need for training on the meaning of questions to allow for further explanations by data collectors or those administering mental health screeners. While gathering accurate responses is critical, relying on data collectors to explain unclear concepts may impact the data quality due to variability in explanations. This finding points to the limitations and applicability of mental health screeners, particularly the PHQ-9, that may have persistent translation and cultural applicability challenges. Although depression is a mental disorder that exists across cultures (Kessler and Bromet 2013), there is evidence to suggest that the way it manifests and is understood may differ (Bhugra and Mastrogianni 2004; Haroz et al. 2017). Instruments for screening for symptoms of mental health that have been adapted and interrogated for validity beyond statistical approaches may be more likely to yield accurate assessments of depression symptoms in both research and clinical settings.

This study has limitations. Interviews were conducted by telephone, which restricted our sample to individuals with telephone access, particularly for participants living with HIV. However, these participants were drawn from the RCCS, which obtains reliable telephone contact information from all participants (even if they do not own a phone), which may have mostly mitigated this concern in our sample. While a small number of participants were referred for further mental health evaluation based on their responses to the screeners, we did not sample people living with HIV based on mental health-related criteria. This may limit our ability to understand differences in item interpretation between individuals with and without symptoms of mental distress. However, sampling on level of education and gender and including both people living with HIV and data collectors/health workers strengthens our ability to comment on how questions may be interpreted by individuals outside of just recipients of screening instruments. Lastly, this study focused on the Luganda language translations of instruments and findings about the content of questions that may not apply to other settings and languages. However, there was overlap in our study with a similar study conducted in Malawi on questions that were challenging for participants to understand (Harrington et al. 2021), suggesting some questions, specifically questions 1 and 6, may need particular attention when translated and phrased even in other languages beyond Luganda. In addition, consideration of whether the PHQ-9 is the most appropriate screener to identify people with symptoms of depression is warranted for practitioners and researchers, depending on the setting.

## Conclusions

The findings from this cognitive interviewing study add to the scant literature that sheds light on why and how mental health instruments may perform well or sub-optimally when used in different languages and contexts. Adaptation and use of instruments that measure mental health outside of the contexts and languages in which they were developed will often require approaches that go beyond assessing instrument validity statistically to also assess meaningfulness and utility, such as through the inclusion of idioms of distress and careful consideration of language and local context as they relate to instrument items.

## Data Availability

The data supporting this study’s findings are available from the corresponding author, NSW, upon reasonable request.

## Impact Statement

This research highlights the importance of culturally relevant mental health screening tools for people living with HIV in Uganda. By adapting and validating the Thinking a Lot Questionnaire, the Patient Health Questionnaire 9 (PHQ-9), and the Hopkins Symptoms Checklist (HSCL) through cognitive interviews, we aim to improve mental health assessment in a region where psychological distress is often overlooked. The refined tools will enable more accurate identification of psychological distress locally, leading to better-targeted interventions and support for people living with HIV. This can enhance treatment adherence, reduce transmission rates, and improve overall health outcomes in Uganda and similar settings. Regionally, this research serves as a model for other African countries facing similar mental health assessment challenges. By emphasizing cultural relevance and local idioms of distress, different regions can adapt their mental health tools to be effective and meaningful for diverse populations. Internationally, this study contributes to the broader understanding of cross-cultural mental health assessment. It underscores the need to validate mental health instruments beyond statistical measures, considering local context and language nuances. This approach can inspire global health organizations and researchers to adopt more inclusive methods when developing and implementing mental health tools in various cultural settings. Ultimately, this research promotes a more nuanced and effective approach to mental health care for marginalized populations, fostering equity in health care access and quality worldwide. By ensuring that mental health screening tools are culturally and contextually appropriate, we pave the way for better mental health outcomes for millions of people living with infectious and chronic illnesses globally.
